# Binding Properties of RNA Quadruplex of SARS-CoV-2 to Berberine Compared to Telomeric DNA Quadruplex

**DOI:** 10.3390/ijms23105690

**Published:** 2022-05-19

**Authors:** Rosario Oliva, Sanjib Mukherjee, Magiliny Manisegaran, Marco Campanile, Pompea Del Vecchio, Luigi Petraccone, Roland Winter

**Affiliations:** 1 Physical Chemistry I—Biophysical Chemistry, Department of Chemistry and Chemical Biology, TU Dortmund University, Otto-Hahn Street 4a, 44227 Dortmund, Germany; sanjib.mukherjee@tu-dortmund.de (S.M.); magiliny.manisegaran@tu-dortmund.de (M.M.); 2 Department of Chemical Sciences, University of Naples Federico II, Via Cintia 4, 80126 Naples, Italy; marco.campanile@unina.it (M.C.); pompea.delvecchio@unina.it (P.D.V.); luigi.petraccone@unina.it (L.P.)

**Keywords:** RNA G-quadruplex, SARS-CoV-2, berberine, ligand binding, hTel G-quadruplex, high pressure

## Abstract

Previous studies suggest that berberine, an isoquinoline alkaloid, has antiviral potential and is a possible therapeutic candidate against SARS-CoV-2. The molecular underpinnings of its action are still unknown. Potential targets include quadruplexes (G4Q) in the viral genome as they play a key role in modulating the biological activity of viruses. While several DNA-G4Q structures and their binding properties have been elucidated, RNA-G4Qs such as RG-1 of the N-gene of SARS-CoV-2 are less explored. Using biophysical techniques, the berberine binding thermodynamics and the associated conformational and hydration changes of RG-1 could be characterized and compared with human telomeric DNA-G4Q 22AG. Berberine can interact with both quadruplexes. Substantial changes were observed in the interaction of berberine with 22AG and RG-1, which adopt different topologies that can also change upon ligand binding. The strength of interaction and the thermodynamic signatures were found to dependent not only on the initial conformation of the quadruplex, but also on the type of salt present in solution. Since berberine has shown promise as a G-quadruplex stabilizer that can modulate viral gene expression, this study may also contribute to the development of optimized ligands that can discriminate between binding to DNA and RNA G-quadruplexes.

## 1. Introduction

The COVID-19 pandemic, which started spreading in late 2019, is caused by a new enveloped RNA coronavirus, named severe acute respiratory syndrome-coronavirus-2 (SARS-CoV-2). In an unparalleled effort, the structural and biochemical properties of SARS-CoV-2 components have been determined in the following months [1,2,3]. Even though vaccines have also been quickly developed and released, including the novel mRNA technology, COVID-19 is still raging worldwide as of spring 2022, pushed by the emergence of a more contagious viral strain.

**Figure 1:**
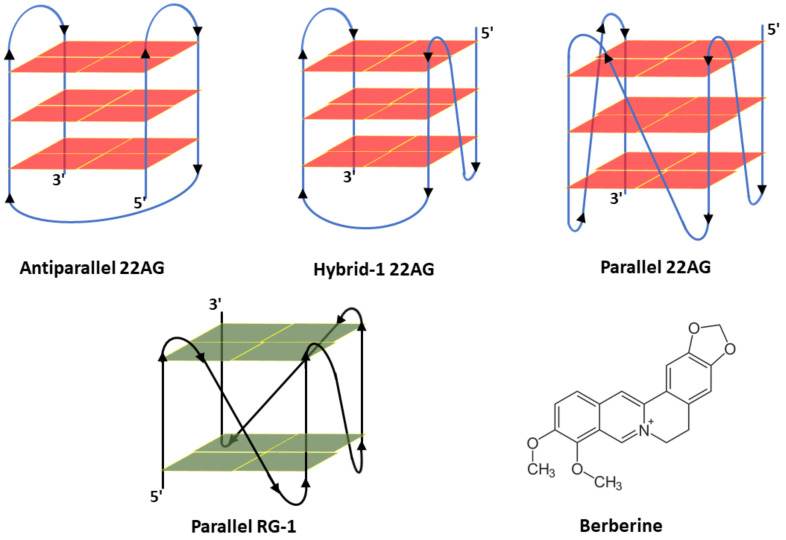
Schematic representation of the possible structures of the human telomeric DNA quadruplex 22AG (antiparallel, hybrid-1, and parallel) and the RNA quadruplex from the genome of SARS-CoV-2, RG-1 (parallel). The chemical structure of the ligand berberine is also shown.

So far, efficient and safe antiviral agent have not been developed [4,5,6,7]. In recent years, non-canonical nucleic acids structures, such as G-quadruplexes, have been spotlighted as promising therapeutic targets [8,9,10,11,12,13,14,15]. G-quadruplexes (G4Qs) are common in guanine-rich regions and are characterized by the stacking of two or more planar arrangements of four guanines (tetrads), which are stabilized by the formation of Hoogsten-type hydrogen bonds and by the interaction with monovalent cations (Na^+^, K^+^) placed in their central canal (Figure 1). G4Qs have received particular attention due to their involvement in the control of gene expression [11,12,13,14,15,16,17]. Dysregulation of G4Q formation and their binding proteins, which assist them in regulating the equilibrium between their structured and unstructured forms, due to mutations or via the alteration of their stability by environmental factors (e.g., by G4Q-stabilization induced by a polypeptide or small-molecule ligand), have been found to contribute to many human pathologies, including neurodegenerative diseases, cancer, and microbial infections [17]. Depending on the orientation of the strands and their loop structures, the structure of G4Qs can be parallel (with four strands in the same orientation), antiparallel (with two strands in opposite orientation with respect to the other two), or hybrid (with one strand in opposite orientation with respect to the others) [15,16,17].

Next to eukaryotes and prokaryotes, G4Q motifs are also considered key elements in regulating the life cycle of viruses [7,8,9,10,18]. Quadruplex folding may preserve the viral genetic material, avoiding its recognition by components of the human immune system. On the other hand, an overstabilization of G4Qs may inhibit the translation of viral proteins by the host’s cellular apparatus. Hence, G4Q-specific compounds could be potential candidates for antiviral agents. Recently, it has been shown that G4Q-forming sequences in SARS-CoV-2 can form G4Q structures in living cells [8,19]. Hence, they would be novel targets for G4Q-specific compounds that could exert antiviral activity. The G4Q-forming sequence RG-1 is located in the coding sequence region of the SARS-CoV-2 nucleocapsid phosphoprotein (denoted N-protein), and the formation of RG-1 has been shown to decrease the amount of N-protein by inhibiting its translation [8]. The presence of G4Qs in infected living cells has also been confirmed. Therefore, stabilization by ligands can induce the downregulation of their expression, hence impairing the maturation and infectivity of viral proteins and paving the way to promising therapeutic strategies.

Several planar ligand molecules, including the plant-derived isoquinoline alkaloid berberine (Berb) (Figure 1), were found to bind strongly to G-tetrads. X-ray diffraction and modelling studies showed that two berberine molecules attach at a time to the two external binding sites of human telomeric DNA (22AG). Several studies have proven that berberine, a common folk medicine for hundreds of years in China, has several biological effects, including antiviral, anti-allergic, and anti-inflammatory properties, and may even help against COVID-19 [20,21,22]. It was shown that berberine is effective against multiple isolates of SARS-CoV-2 and may be a promising orally administered therapeutic candidate against the disease that could be considered for further efficacy testing. Though interfering with capsid protein-viral RNA interactions and interference with cellular pathways to reduce SARS-CoV-2 replication have been proposed as possible scenarios, the molecular mechanism of its action is still largely *terra incognita*.

A prerequisite for the development of G4Q-related antiviral drugs against COVID-19 is a detailed knowledge of the structure and stability of the G4Q-ligand complex. In this study, we set out to explore the binding characteristics of berberine to RNA RG-1 G4Q of the N-gene of SARS-CoV-2 and compare it with human telomeric DNA G4Q, 22AG. Berberine is a planar molecule with an extended π-delocalized system. Having a partial positive charge on N7 (Figure 1), it is able to bind to 22AG and inhibit telomerase activity [22,23,24,25]. Indeed, berberine was shown to bind to 22AG through stacking interactions with the terminal G-planes [24], and this constituted the starting point to elucidate the interaction mechanism of berberine with RG-1. Telomerase, a ribonucleoprotein reverse transcriptase enzyme, is involved in the cell’s life cycle. The enzyme adds a repeat sequence to the 3′ end of telomeres, protecting the end of the chromosome from DNA damage. It is active in gametes and overexpressed in cancer cells, but is normally absent from somatic cells, or present at very low levels. Using a variety of biophysical techniques, including isothermal titration calorimetry (ITC), circular dichroism (CD) and fluorescence spectroscopy (including Job’s plots), a full characterization of the binding thermodynamics and the associated conformational and hydration changes upon binding could be achieved and is compared to that of human telomeric G4Q 22AG. Complementary pressure-dependent measurements revealed changes in packing density and hydration upon complex formation. Considerable differences in binding properties and conformational signatures between the RNA and DNA G4Qs were found. Since berberine has shown promise as a G-quadruplex stabilizer that can modulate gene expression via its interaction with G4Qs of SARS-CoV-2, this study may help to develop optimized ligands for intervention.

## 2. Results

### 2.1. Binding of Berberine to 22AG and RG-1 at Different Solution Conditions

**Figure 2:**
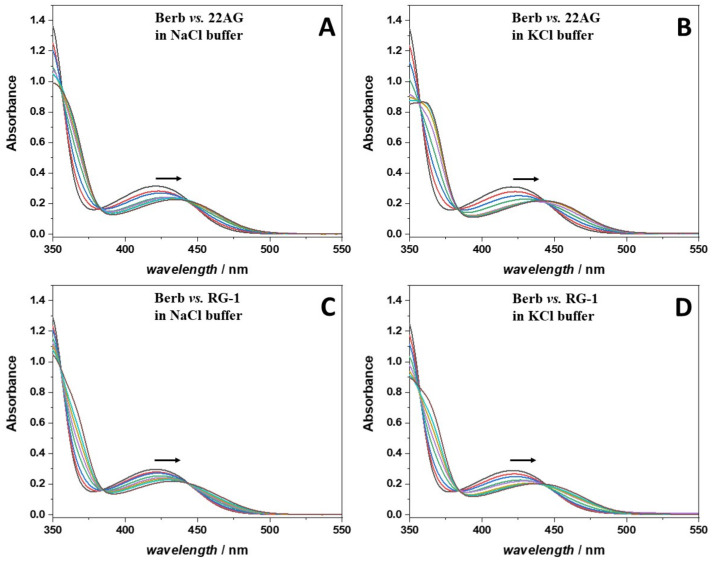
UV/Vis absorption spectra of a 250 µM berberine (Berb) solution at increasing concentrations of: (**A**) 22AG in Na^+^ containing buffer; (**B**) 22AG in K^+^ containing buffer; (**C**) RG-1 in Na^+^ containing buffer, and (**D**) RG-1 in K^+^ containing buffer. The concentration of the quadruplexes (22AG or RG-1) was: 10 µM (red lines), 20 µM (blue lines), 40 µM (green lines), 60 µM (violet lines), 80 µM (dark yellow lines), 100 µM (cyan lines), and 150 µM (brown lines). The spectra of berberine in the absence of any quadruplex are reported as black lines. All spectra were recorded at 25 °C using a 0.3 cm path length quartz cuvette in 30 mM Tris buffer, pH 7.4, with the addition of 100 mM of NaCl or KCl. The arrows indicate the direction of the shift of the absorption maximum in the berberine spectrum with increasing concentrations of G4Qs.

We first employed UV/Vis spectrophotometry to study the binding of the ligand berberine to the human telomeric (22AG) and the SARS-CoV-2 RG1 quadruplex at different solution conditions. To this end, UV/Vis spectra of berberine in the absence and in the presence of increasing amounts of the G4Qs (22AG, RG-1) in 30 mM Tris-HCl, pH 7.4, with the addition of 100 mM KCl or NaCl were recorded. The decision to use these salt concentrations was motivated by the fact that the structure of the 22AG sequence was well characterized under in these conditions, adopting a well-defined antiparallel conformation in the presence of sodium cations, and a hybrid-1 conformation in the presence of potassium cations (see Section 2.2 for a deeper discussion). The berberine concentration was 250 µM, and the concentration of the G4Qs was varied between 0 µM and 150 µM. All the spectra are reported in Figure 2.

The UV/Vis spectrum of berberine is characterized by an absorption band centered at about 421 nm when dissolved in aqueous solution. Upon addition of the G4Qs, a clear shift of the maximum was observed, associated with a decrease of the absorbance. A well-defined isosbestic point at 443 nm was present in all cases, which is a clear indication that berberine is able to interact with both G4Qs (22AG and RG-1) in both media (Na^+^ and K^+^ containing buffers).

**Figure 3:**
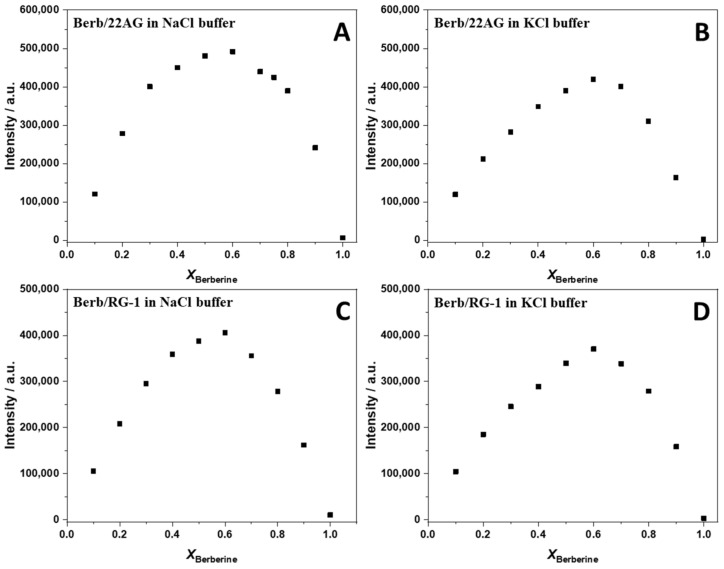
Job’s plot for the Berb/22AG (panel (**A**,**B**)) and Berb/RG-1 (panel (**C**,**D**)) systems obtained from fluorescence spectroscopy in 30 mM Tris buffer, pH 7.4, with the addition of 100 mM NaCl or KCl. The total concentrations ((Berb) + (G4Qs)) were 80 µM, 60 µM, 60 µM, and 50 µM for the experiments shown in panel (**A**–**D**), respectively. The excitation wavelength was set at 443 nm. The fluorescence intensity was recorded at the maximum of emission (536 nm). All the experiments were carried out at 25 °C and ambient pressure.

The strength of interaction of the ligand to the G4Q can be quantitatively described by the binding (or affinity) constant, denoted as *K*_b_, which is defined as the ratio of the concentration of the complex to the product of the free ligand and G4Q concentrations [26]. In order to evaluate *K*_b_, a titration experiment was performed where the concentration of one of the two interacting partners was kept constant, while the other one was varied in a suitable range. The experimental data were fitted with an appropriate binding model, which describes how (e.g., independent sites) and how many ligands are bound to one biomacromolecule. To apply a suitable binding model, it is of fundamental importance to know in advance the stoichiometry of the complex formed. To this end, we applied to method of the continuous variation, also known as Job’s plot, to the formation of the complex between berberine and 22AG or RG-1 in both salt solutions. The experiments were performed preparing a series of solutions such that the total concentration of the interacting partners was kept constant while their mole fraction was varied [27]. Then, the fluorescence emission of these solutions was recorded by exciting at 443 nm (at the isosbestic point observed in the UV/Vis spectra, where both the free berberine and the complex have the same extinction coefficient, see Figure 2) and collecting the emission at *λ*_max_ (~536 nm). Fluorescence spectroscopy was chosen to follow the binding because berberine is almost not fluorescent in aqueous solution, whereas a strong fluorescence increase is observed upon binding to G4Qs [28,29]. Thus, the fluorescence intensity is directly related to complex formation. Abrupt changes in the slope of the Job’s plots indicate the stoichiometry of the complex formed. The Job’s plots at ambient conditions (*T* = 25 °C, *p* = 1 bar) are reported in Figure 3.

An inspection of the Job’s plots depicted in Figure 3 reveals for all cases an inflection point centered at about x_Berberine_ = 0.6, suggesting that two berberine molecules are bound to one G4Q. In an ideal case, the inflection point for such stoichiometry should be located at x_Berberine_ = 0.67. However, the exact position and the shape of the Job’s plot depends also on the magnitude of the binding constant and the total ligand concentration employed (see the ESI with Supplementary Figures S1–S3 for an in-depth discussion of Job’s plots, where also simulations for different cases are shown). Simulations taking the magnitude of *K*_b_ and total ligand concentration into account revealed that in fact a 2:1 Berb:G4Q complex is formed for both the 22AG and RG-1 G4Q, and the stoichiometry did not depend on the solution conditions, i.e., on the type of salt.

**Figure 4:**
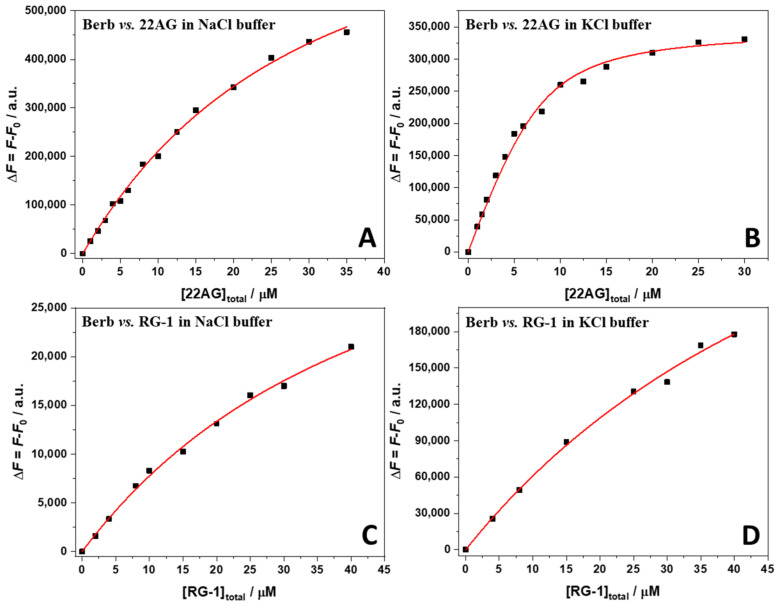
Binding isotherms for the Berb/22AG (panel (**A**,**B**)) and Berb/RG-1 (panel (**C**,**D**)) systems obtained from fluorescence spectroscopy in 30 mM Tris buffer, pH 7.4, with the addition of 100 mM NaCl or KCl. The berberine concentration was 15 µM and the concentration of G4Qs was varied between 0 µM and 40 µM. The berberine was excited at 443 nm, the emission was collected at the maximum of emission, at 536 nm. The red lines represent the best fit of the experimental data according to an equivalent and independent binding sites model. According to the Job’s plot data, the stoichiometry was 2:1 (Berb:G4Q) in all cases. All the experiments were performed at 25 °C and 1 bar.

Now that the stoichiometries of the complexes formed are known, it was possible to determine the values of the binding constant through fluorescence titration experiments. To this end, a 15 µM solution of berberine was titrated with a solution of 22AG or RG-1 in the concentration range 0–40 µM. The experimental data could all be well fitted using an equivalent and independent binding sites model [30], i.e., it was assumed that the two berberine molecules are bound with the same affinity. Figure 4 shows all binding isotherms obtained at ambient conditions (*T* = 25 °C, *p* = 1 bar). The values of the binding constants, *K*_b_, are collected in Table 1.

The data reported in Table 1 show that the binding constant, *K*_b_, for the complex formation of the human telomeric sequence (22AG) with berberine is strongly dependent on the initial conformation of 22AG. The initial conformation of 22AG was antiparallel in the presence of Na^+^. Conversely, 22AG adopts a hybrid-1 conformation in the presence of K^+^ (see the CD experiments below). Berberine has the highest affinity for 22AG in the presence of K^+^ cations in solution. In the Na^+^ containing buffer, a one order of magnitude decrease of the *K*_b_ value was detected. A different scenario was observed for the RNA RG-1 sequence: the binding constant is smaller, and it shows a rather low dependence on the solution conditions, i.e., on the type of cation.

**Figure 5:**
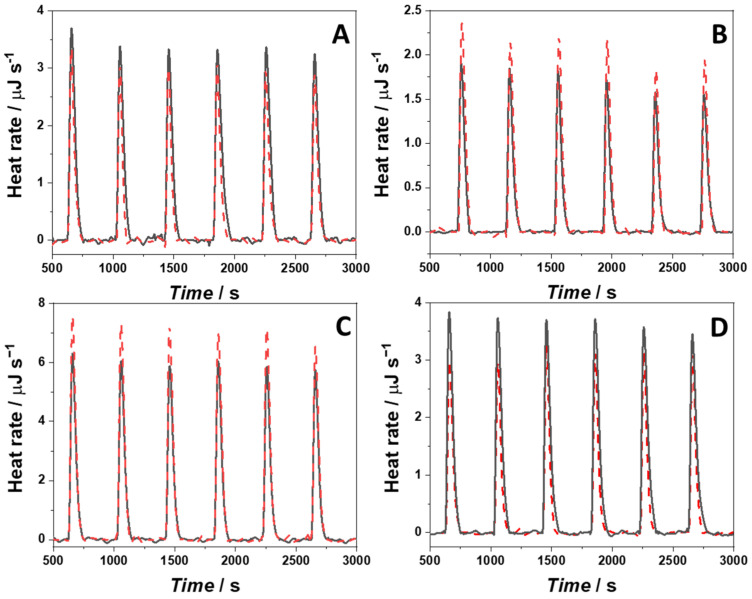
ITC traces (black lines) obtained from the titration of a solution of (**A**) 22AG in the presence of 100 mM NaCl, (**B**) 22AG in the presence of 100 mM KCl, (**C**) RG-1 in the presence of 100 mM NaCl, and (**D**) RG-1 in the presence of 100 mM KCl, with a solution of berberine. The red dashed lines represent the heat peaks obtained from the G4Q dilutions in the respective buffers. All the experiments were carried out in 30 mM Tris-HCl buffer, pH 7.4, at the temperature of 25 °C.

To yield further thermodynamic insights into the interaction process between berberine and the two different G4Qs, isothermal titration calorimetry (ITC) experiments were carried out. The ITC experiments allowed us to determine also the enthalpy change upon binding, i.e., the standard binding enthalpy, Δ*H*°_b_. Except for the Berb/22AG system in K^+^ containing buffer, which exhibits a suitably high binding constant, ITC cannot be used in the conventional way. This is because the binding constants are too low and, thus, very high concentrations should be used to obtain conventional sigmoidal binding curves. However, the binding enthalpy can still be determined by injecting a small amount of G4Qs into the calorimetry vessel where a large excess of berberine is present. Since the berberine concentration was in large excess with respect to the G4Q concentration, similar heat peaks were obtained at each step of the titration. The heat rates recorded were normalized by the moles of bound G4Qs that can be easily calculated from the values of the binding constants. Figure 5 reports the ITC traces obtained from the titration of a berberine solution (in NaCl or KCl containing buffer, at *T* = 25 °C) for a solution of 22AG or RG-1. The corresponding enthalpy changes are reported in Table 1, together with the other thermodynamic parameters determined, i.e., the standard Gibbs (free) energy of binding, Δ*G*°_b_, and the binding entropy term *T*Δ*S*°_b_.

The binding enthalpy for complex formation between berberine and 22AG in the presence of 100 mM NaCl was found to be negative, Δ*H*°_b_ = −15.5 kJ mol^−1^, i.e., the binding is an exothermic process. The entropic contribution to binding, *T*Δ*S*°_b_, is positive (10.5 kJ mol^−1^), i.e., is favorable for binding as well. Surprisingly, for the same system in the presence of 100 mM KCl, Δ*H*°_b_ is positive (19.5 kJ mol^−1^). With a positive *T*Δ*S*°_b_ of 52.4 kJ mol^−1^, the Gibbs energy of binding is still more negative (Δ*G*°_b_ = −32.9 kJ mol^−1^), rendering the binding process entropy-driven. For the SARS-CoV-2 quadruplex RG-1, an opposite behavior was observed with respect to the enthalpy change. Δ*H*°_b_ = 75.9 kJ mol^−1^ is positive for the complex formation when NaCl is dissolved in the medium, and *T*Δ*S*°_b_ = 101.3 kJ mol^−1^. Since both Δ*H*°_b_ and *T*Δ*S*°_b_ are positive, the complex formation is entropy-driven. In the presence of potassium cations, instead, both the enthalpy and entropy changes are negative (Δ*H*°_b_ = −92 kJ mol^−1^, *T*Δ*S*°_b_ = −66.9 kJ mol^−1^), rendering the complex formation enthalpy-driven. Due to enthalpy-entropy compensation, the Gibbs energies of complex formation and binding constants are hence of the same magnitude as for the interaction of berberine with 22AG in NaCl solution.

### 2.2. The Conformational Behavior of 22AG and RG-1 in Complex with Berberine

**Figure 6:**
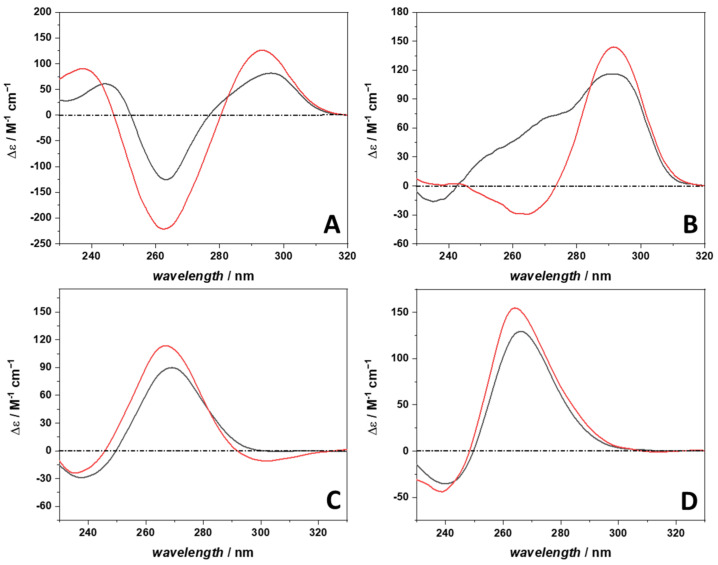
Circular dichroism (CD) spectra of the 22AG quadruplex in the absence (black spectra) and in the presence (red spectra) of berberine (22AG:Berb = 1:10) in (**A**) 30 mM Tris, 100 mM NaCl, pH 7.4, and (**B**) 30 mM Tris, 100 mM KCl, pH 7.4. In the bottom panels, the CD spectra for the RG-1 sequence in the absence (black spectra) and in the presence (red spectra) of berberine (22AG:Berb = 1:10) are reported for the measurements carried out in (**C**) 30 mM Tris, 100 mM NaCl, pH 7.4, and (**D**) 30 mM Tris, 100 mM KCl, pH 7.4. The path length of the cuvette was 0.1 cm. The temperature was set to 25 °C.

To help explain the different thermodynamic signatures of complex formation of the different systems, the conformation of the 22AG and RG-1 quadruplexes in the absence and in the presence of berberine was determined by means of circular dichroism (CD) spectroscopy [31,32]. Figure 6 depicts the CD spectra of 30 µM 22AG and RG-1 in the absence and in the presence of 300 µM berberine, i.e., for the molar ratio G4Q:Berb = 1:10.

The CD spectrum of 22AG in the presence of 100 mM NaCl (Figure 6, panel A) is characterized by a positive band centered at about 295 nm. A negative band is present at ~260 nm, followed by a band at 245 nm. These spectral features are indicative of a quadruplex adopting an antiparallel conformation [31,33]. Upon berberine binding, the general spectral features of 22AG remain the same, indicating that the conformation of 22AG is still antiparallel. However, it is important to note that the intensities of the bands are not the same, suggesting that small (local) conformational changes took place (e.g., in the loop region). Instead, the 22AG sequence in the K^+^ containing medium adopts mainly a hybrid-1 conformation (Figure 6, panel B). The CD spectrum shows a positive band around 292 nm, which is followed by a small positive band around 270 nm. In addition, a weak negative band around 235 nm is seen. Upon complex formation with berberine, a clear conformational change was observed. The CD spectrum shows features of an antiparallel conformation (positive band around 295 nm and a negative one around 265 nm). However, with respect to the CD spectrum in Na^+^ containing buffer, the band intensities are different, indicating that the two conformations are not fully equivalent.

For RG-1 in the presence of Na^+^ (Figure 6, panel C), we observed an intense positive band around 270 nm and a weak negative band around 240 nm, indicating formation of a parallel conformation, in agreement with literature data [10]. When berberine is present in solution, the general spectroscopic features of a parallel conformation are preserved. However, small but significant shifts of the maximum and minimum were observed. In addition, a weak negative band around 300 nm was detected. These observations point to some local conformational changes taking place upon berberine binding. In the K^+^ containing solution medium (Figure 6, panel D), the position of CD bands suggests that RG-1 adopts a parallel conformation as well. Upon complex formation, only small changes (regarding the intensity and shifts of the maximum and minimum) were observed, suggesting that RG-1 is adopting a parallel conformation also in the complexed form. Small conformational changes cannot be excluded, however. Although the CD spectra of RG-1 in Na^+^ and K^+^ containing buffer (black lines in Figure 6, panels C and D) exhibit the same general features, the intensities of the bands are different. In particular in the presence of Na^+^, the positive band is less intense with respect to that in K^+^, indicating that the RG-1 is not fully folded or, most likely, a population of (partially) unfolded conformers coexist, as also previously observed in single-molecule FRET experiments [34]. Indeed, the spectrum of the fully unfolded RG-1 recorded at 75 °C shows a positive band around 270 nm, which is less intense with respect to the same band at 25 °C (see Supplementary Figure S4).

### 2.3. Probing Volumetric and Hydration Properties upon Complex Formation between Berberine and G4Qs Using Pressure Modulation Spectroscopy

**Figure 7:**
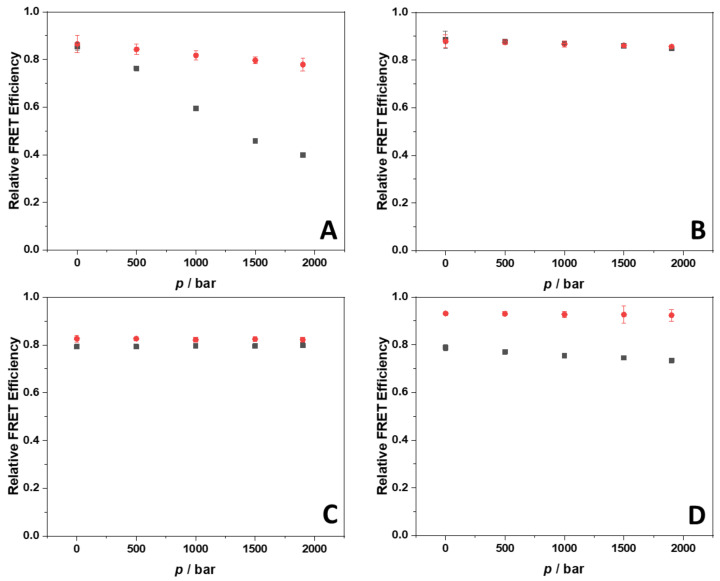
Relative FRET efficiency vs. pressure plots for the dually labeled 22AG (panels (**A**,**B**)) and RG-1 (panels (**C**,**D**)) quadruplex forming sequences in the absence (black squares) and in the presence (red circles) of berberine in 30 mM Tris buffer, pH 7.4, with the addition of 100 mM NaCl (panels (**A**,**C**)) and 100 mM KCl (panels (**B**,**D**)). The concentration of the labeled G4Q sequences was 2 µM, the concentration of berberine was 200 µM. All the experiments were performed at the temperature of 25 °C. Where not visible, the error bars are smaller than the symbol size.

The application of high hydrostatic pressure (HPP) is a powerful tool to study the volumetric properties of reactions including complex formation [35]. However, before being able to explore the effect of HHP on the strength of interaction as described by *K*_b_ between berberine and the G4Qs, it is mandatory to verify that the structure of 22AG and RG-1 and in complex with berberine is not changing. To this end, we employed the high-pressure FRET methodology using dually labeled 22AG and RG-1. Both sequences were labeled with carboxyfluorescein (FAM) at the 3′ position and with 5-(and-6)-carboxytetramethylrhodamine (TAMRA) at the 5′ position which act as FRET pair. The efficiency of the energy transfer is sensitive to the distance between the two fluorophores which, in turn, depends on the conformation adopted by the G4Qs. When the two fluorophores are close to each other, as in the case when the G4Q is folded, high values of the efficiency are observed. Conversely, lower values are detected upon unfolding of the G4Qs. Figure 7 shows plots of the relative FRET efficiency, *E*_rel_, as a function of pressure for 22AG and RG-1 in the absence and in the presence of berberine in the two different salt solutions.

At ambient pressure (1 bar) and 25 °C, a very high *E*_rel_ value was detected (~0.85) for the 22AG sequence in the Na^+^ containing buffer, indicating that the sequence is folded. According to the CD spectroscopy data, the 22AG sequence adopts an antiparallel conformation at this condition. In our previous work, we recorded a similar value (*E*_rel_ = 0.81) for the same sequence in 60 mM Na^+^ containing buffer [33]. Increasing pressure leads to a marked decrease of *E*_rel_ (*E*_rel_ ≈ 0.40 at 1900 bar), indicating that the quadruplex structure is not pressure stable at these solution conditions, i.e., pressurization up to about 2 kbar leads to a significant population of unfolded states, which is in agreement with earlier findings [33]. In the presence of berberine, at 1 bar, a value of *E*_rel_ similar to that observed in its absence was observed, suggesting, in agreement with the CD data, that the antiparallel conformation is preserved upon berberine binding. Remarkably, the application of pressure to the complex had only a minor effect, indicating that the 22AG has gained drastic pressure stability upon complex formation. In the presence of K^+^ ions, at 1 bar, the FRET efficiency of the 22AG sequence is again very high, indicating a folded conformation, which, according to the CD data, is mainly hybrid-1. Upon application of pressure up to 1900 bar, no changes in the FRET efficiency were observed, indicating that the quadruplex structure adopted in the presence of K^+^ is pressure stable. Upon complex formation with berberine, an *E*_rel_ value close to that one in the absence of ligand was detected. According to the CD data, the 22AG should be folded in an antiparallel conformation now, which is obviously also very pressure stable in the presence of bound berberine.

The same experiments were then carried out for the RG-1 sequence. Please note that since the 22AG and RG-1 sequences are of different lengths (22 vs. 15 n.a. residues, respectively), a direct comparison of the FRET efficiencies is not possible. For the RG-1 sequence at 1 bar and in the presence of Na^+^, the detected efficiency is very high (*E*_rel_ ≈ 0.79), which is indicative of a folded structure, a parallel conformation according to the CD data. Application of HHP does not cause any changes in the efficiency, revealing that the structure is not sensitive to pressure. This result is in sharp contrast to that obtained for the 22AG sequence, which showed little pressure stability for its antiparallel structure in Na^+^ solution. Upon complexation with berberine, the value of *E*_rel_ slightly increased, only. This indicates that the conformation of RG-1 is not dramatically affected by the interaction process and, according to the CD data, essentially retains its parallel conformation. Application of HHP has no effect on the conformational behavior of the quadruplex, revealing that the RG-1/Berb complex is pressure stable. In the presence of K^+^, the *E*_rel_ value is similar to that observed in the presence of Na^+^. Upon complex formation with berberine, a significant increase of the *E*_rel_ value was observed, however. The CD data suggest that the RG-1 is mainly folded in a parallel fashion. Hence, the increase of *E*_rel_ could be ascribed to some small conformational change of RG-1 upon binding to berberine, as also suggested by the small differences in the CD spectra (Figure 5).

The 22AG sequence labeled with FAM and TAMRA is widely used, its usefulness for conformational studies approved, and several applications are reported in the literature [33,36,37]. This is not yet the case for the RG-1 sequence. For this reason, as a control experiment, we performed the same experiment for RG-1 in neat buffer conditions (no salts) as a function of temperature and recorded complementary CD spectroscopic data (see ESI, Supplementary Figure S4). The results obtained validate our findings, indicating that the two attached fluorophores have no detectable influence on the conformational behavior of the RG-1 sequence.

**Figure 8:**
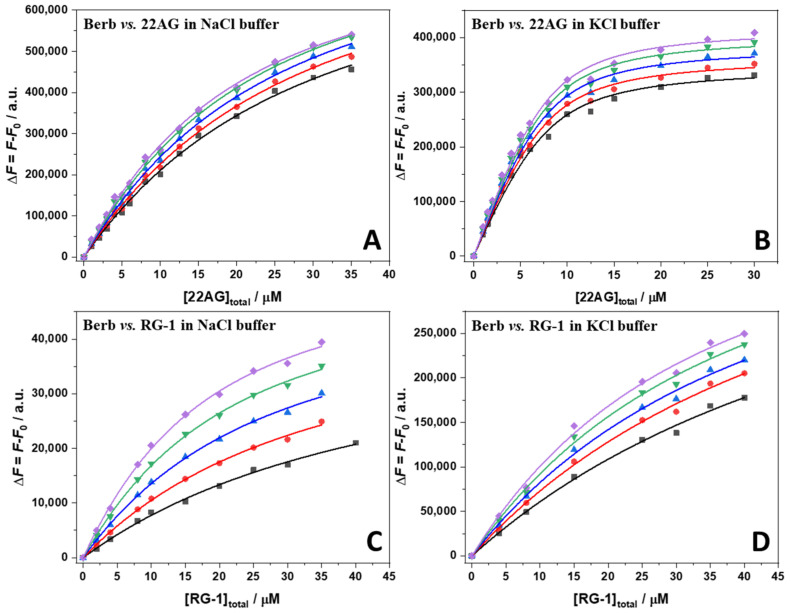
Binding isotherms for the Berb/22AG (panel (**A**,**B**)) and Berb/RG-1 (panel (**C**,**D**)) systems obtained from high pressure fluorescence spectroscopy in 30 mM Tris buffer, pH 7.4, with the addition of 100 mM NaCl or KCl at the temperature of 25 °C and pressures of: 1 bar (black squares), 500 bar (red circles), 1000 bar (blue triangles), 1500 bar (green reversed triangles), and 1900 bar (violet diamonds). The berberine concentration was 15 µM, the concentration of the G4Qs was varied between 0 µM and 40 µM. The berberine was excited at 443 nm, whereas the emission was collected at the maximum of emission, at 536 nm. The continuous lines represent the best fit of the experimental data according to an equivalent and independent binding sites model using the stoichiometries reported in Table 2.

Finally, we explored the effect of HHP on the interaction strength, i.e., *K*_b_ value, of the different G4Q systems, which is straightforward for those systems that are pressure stable. In those cases, a change in binding stoichiometry is not envisaged. However, we observed that the 22AG structure in the presence of 100 mM NaCl changes its conformation upon pressurization, leading to unfolding of the quadruplex. In such case, a change of the binding stoichiometry is expected to occur. Supplementary Figure S5 shows the Job’s plot for the Berb/22AG system in the presence of 100 mM NaCl at pressures of 500, 1000, 1500 and 1900 bar. At 500 bar, as well as at 1000 bar, the inflection point is centered at about *x*_Berberine_ = 0.6, suggesting that two berberine molecules are still bound to one 22AG, as in the case at 1 bar. Increasing the pressure up to 1500 bar and even more pronounced at 1900 bar, the inflection point is shifting to *x*_Berberine_ = 0.7, indicating a change in the stoichiometry from 2:1 to 3:1 (Berb:22AG), which is most likely due to an increase of the number of bound berberine molecules in the pressure-unfolded state structure of the 22AG. Figure 8 depicts all the binding isotherms measured at *T* = 25 °C in the pressure range from 1 bar to 1900 bar. The values of the binding constants determined are collected in Table 2.

For the complex formation between berberine and 22AG in the presence of 100 mM NaCl, the application of pressure leads to a small increase of *K*_b_, which increases from 0.37 ∙ 10^5^ M^−1^ at 1 bar to 0.50 ∙ 10^5^ M^−1^ at the highest pressure applied (1900 bar). A similar scenario was observed in the presence of K^+^. Hence, pressure favors complex formation slightly for the system Berb/22AG in both media. Owing to partial pressure-induced unfolding, a change in stoichiometry is observed at high pressures in the case of the Na^+^ containing solution. Surprisingly, application of pressure has a much more prominent effect in case of the complexation reaction of berberine with the RG-1 quadruplex. In Na^+^ containing buffer, the *K*_b_ increased from 0.28 ∙ 10^5^ M^−1^ at 1 bar to 0.77 ∙ 10^5^ M^−1^ at 1900 bar. A similar situation was observed in the presence of K^+^, the increase of *K*_b_ upon pressurization being less pronounced compared to that in the presence of Na^+^, however.

Measurement of the pressure dependence of *K*_b_ allows to determine the volume change accompanying of the binding reaction, Δ*V*_r_, according to [35]:

(1) $${\left[\frac{\mathrm{dln}\left(K_{b}\right)}{dp}\right]}_{T}=-\frac{\Delta V_{r}}{RT}$$

Δ*V*_r_ is defined as the partial molar volume difference between the complex state (berberine bound to G4Q) and the sum of the partial molar volumes of the ligand and the G4Q molecule in their uncomplexed state. It is important to note that Δ*V*_r_ includes both the volume change for the binding event itself (including hydration changes) and the volume change for an accompanying conformational transition, if there is one.

**Figure 9:**
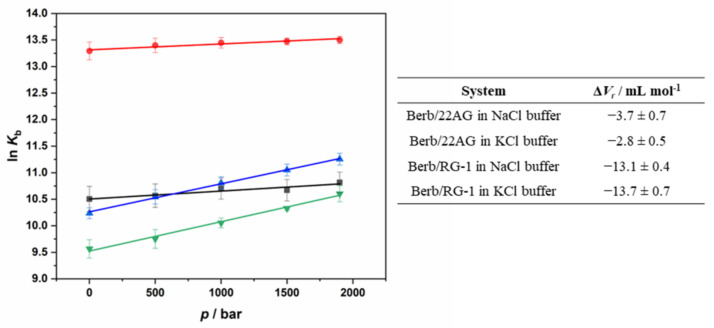
Left: Plots of the natural logarithm of *K*_b_ vs. pressure (in bar) for the complex formation between berberine and 22AG in the presence of 100 mM NaCl (black squares) and 100 mM KCl (red circles). The pressure dependence of ln(*K*_b_) for the complex formation between berberine and RG-1 is also shown in the same graph: in the presence of 100 mM NaCl (blue triangles) and in the presence of 100 mM KCl (reversed green triangles). The evaluation of the reaction volume, Δ*V*_r_, was determined from the slope of the plots. Right: Reaction volumes, Δ*V*_r_, for the complex formation between berberine and the G4Qs (22AG and RG-1) for the reported solution conditions (30 mM Tris-HCl, pH 7.4, with the addition of 100 mM NaCl or 100 mM KCl) at the temperature of 25 °C.

Figure 9 depicts the plots of ln(*K*_b_) vs. *p* for the complex formation between berberine and the two different G4Qs in Na^+^ and K^+^ containing buffers and reports the Δ*V*_r_ values determined from the slope. The reaction volumes are all negative, indicating that higher pressures favor the formation of the complex. According to Le Châtelier’s principle, increasing pressure favors the state that occupies the smallest possible volume. Thus, negative Δ*V*_r_ values reveal that the partial molar volume of the complex formed by berberine and the G4Qs is smaller compared to the sum of the partial molar volumes of berberine and the G4Qs. It is important to note that for the Berb/22AG system, the Δ*V*_r_ values are similar and very small, indicating that the formation of the complex is only slightly favored by pressure. Conversely, for RG-1, Δ*V*_r_ is significantly more negative (~−13 mL mol^−1^), suggesting that interaction of berberine with RG-1 leads to a significant compaction of the structure.

## 3. Discussion

Using a variety of biophysical techniques, a full characterization of the binding thermodynamics and the associated conformational and hydration changes upon binding of berberine to the RNA G4Q RG-1 could be achieved and the data are compared to those of human telomeric DNA G4Q 22AG. The binding characteristics were explored applying fluorescence and circular dichroism spectroscopy as well as isothermal titration calorimetry. In addition, pressure-dependent experiments were carried out to disentangle the volumetric properties upon complex formation, which inform about packing and hydration changes.

The reported data indicate that berberine is able to interact with both quadruplexes and that two berberine molecules are bound to one G4Q. We found that the strength of the interaction with the telomeric DNA 22AG sequence depends on the initial conformation adopted by the DNA (antiparallel and hybrid-1 in the presence of Na^+^ and K^+^, respectively), but not in the case of the viral RNA RG-1, which adopts a parallel structure under both solution conditions. For 22AG, the *K*_b_ value was found to be one order of magnitude higher in the presence of 100 mM KCl (5.9 ± 1.0 × 10^5^ M^−1^) compared to that in 100 mM NaCl (3.7 ± 0.9 × 10^4^ M^−1^). For the RG-1, the binding constants are similar (2.8 ± 0.3 × 10^4^ M^−1^ in NaCl, 1.48 ± 0.3 × 10^4^ M^−1^ in KCl) for both solution conditions. These observations can be rationalized considering that 22AG adopts a different conformation, an antiparallel one in NaCl and a hybrid-1 one in KCl. Upon berberine binding, 22AG adopts an antiparallel conformation in the presence of KCl, whereas no marked conformational changes of the 22AG were observed in the presence of NaCl. Thus, the strength of interaction with berberine is found to depend on the initial conformation of the quadruplex. Conversely, the RG-1 sequence adopts a parallel conformation in both cases, in the ligand-free and the ligand-bound state.

Berberine is an aromatic molecule formed by five fused rings (Figure 1), rendering berberine a rigid molecule that is able to interact with nucleic acid bases via π-stacking interactions, such as with the G-planes of quadruplexes [23]. The four oxygen atoms of berberine are capable of forming hydrogen bonds, and the nitrogen atom N7 renders berberine positively charged, thus being capable to establish electrostatic interactions with the phosphate groups of the nucleic acids or with the oxygen of the carbonyl group of guanines.

The Job’s plot data suggest that two berberine molecules are bound to one 22AG that adopts an antiparallel conformation. In this conformation, one G-plane is surrounded by two short loops enriched of thymine residues. Instead, the opposite G-plane has one rigid loop, only (Figure 1). Thus, the accessibility to the two planes could be different. Another possibility is that two berberine molecules are bound on the same plane. Such a scenario has been supported by crystal structure data of the complex formed by berberine and the tel23 sequence, where it was shown that first a stoichiometry higher than 1:1 is possible and, secondly, that two berberine molecules can be located on the same plane [24,38]. Which binding mode prevails in the complex formation process is currently not known.

The RG-1 sequence, instead, adopts a parallel conformation, which is essentially preserved upon berberine binding, in both salt media. Our data clearly show that also in this case, two berberine molecules are bound to one quadruplex. For the parallel conformation, both G-planes are equally accessible. Thus, both possible binding modes, i.e., one berberine molecule on each plane or two molecules bound to the same plane, are possible.

ITC experiments were carried out to characterize the energetics of complex formation. Remarkably, it was found that for 22AG, the binding enthalpy is negative in the presence of Na^+^ cations, revealing the formation of favorable non-covalent interactions (probably mainly via π-stacking). Conversely, *T*Δ*S*°_b_ is positive, which could be related to the release of water molecules surrounding the interacting partners upon binding, possibly compensating a possible loss of conformational entropy of the 22AG molecule upon binding. In the K^+^ containing solution, the enthalpy change is positive (Table 1). This could be the case if formation of non-covalent interactions cannot compensate for an unfavorable contribution deriving from unbinding of hydration waters upon binding. This would be in line with the pronounced increase of binding entropy. However, the changes in binding thermodynamics may also derive from conformational changes upon berberine binding. In fact, in KCl solution, a conformational change from the hybrid-1 to the antiparallel conformation takes place upon binding of the ligand. From unfolding experiments, it can be derived that the enthalpy change for the transition from hybrid-1 to the antiparallel conformation is positive (~13 kJ mol^−1^) [39]. Hence, the endothermic enthalpy change recorded for 22AG in KCl solution could in fact derive from a conformational change of the G4Q imposed by berberine binding.

Surprisingly, for RG-1 the binding scenario is entirely different. In the K^+^ containing buffer, the enthalpy change is strongly negative upon binding of berberine (Δ*H*°_b_ = −92.2 kJ mol^−1^), and also the entropy change upon binding is negative (*T*Δ*S*°_b_ = −66.9 kJ mol^−1^). These results indicate that the major contribution to the energetics of complex formation is due to enthalpic stacking interactions established between the G-tetrades of RG-1 and berberine. Upon binding, a loss of conformational entropy of the quadruplex can justify the negative value of the binding entropy, which would be consistent with the observed increase of *E*_rel_ and the small changes in the CD spectra observed upon complex formation. In the presence of Na^+^ ions, the scenario is more intricate. Both the binding enthalpy and entropy are positive (Δ*H*°_b_ = 75.9 kJ mol^−1^, *T*Δ*S*°_b_ = 101.3 kJ mol^−1^). These results are in sharp contrast to the complex formation in KCl solution, where both thermodynamic parameters take on negative values (Δ*H*°_b_ = −92.2 kJ mol^−1^, *T*Δ*S*°_b_ = −66.9 kJ mol^−1^). Breaking of H-bonds upon release of a significant amount of bound water molecules upon ligand binding would contribute positively to both the enthalpy and entropy change. However, a more likely explanation may be found in the details of the conformational dynamics of RG-1. It was previously shown that not all RG-1 molecules in Na^+^ solution are fully folded in a parallel conformation [34]. Single-molecule FRET data are rather consistent with a significant fraction of (partially) unfolded (~50%) states also being present. Berberine binding to the (partially) unfolded conformation could in fact lead to a loss of conformational entropy which is accompanied by a significant release of water molecules, which would be in line with the thermodynamic signatures observed.

The pressure-axis experiments reported above showed that the binding constant of berberine to 22AG increases slightly with increasing pressure, which is characterized by a small negative volume change (~−3 mL mol^−1^) in both salt solutions. Beyond several hundred bars, the antiparallel structure of 22AG starts to unfold in Na^+^ solution. Interestingly, the binding of berberine strongly stabilizes the antiparallel structure of 22AG towards pressure-induced unfolding. Inevitably, the reaction volume reported in Figure 9 should include both the binding volume, Δ*V*_b_, and the conformational transition volume, Δ*V*_conf_, i.e., Δ*V*_r_ = Δ*V*_b_ + Δ*V*_conf_. The volume change for the conformational change from the unfolded to the antiparallel conformation can be estimated to be about 56 mL mol^−1^ (during folding of a G4Q structure, generally a positive volume change is observed [40,41,42,43], which is essentially due to a release of hydration water of nucleic acids and the cations embedded in the inner canal of the quadruplex structure). Thus, the “pure” binding volume may be coarsely estimated to be around −59.7 mL mol^−1^, slightly overcompensating the positive volume change due to the conformational transition. Further, a change in the stoichiometry was observed at 1500 and 1900 bar where three berberine molecules are bound to one 22AG, now. Exposing more potential binding sites upon pressure-induced unfolding prompts the binding of one additional berberine molecule on average. In the presence of KCl, the 22AG adopts mainly a hybrid-1 conformation, which is pressure stable. Upon binding to berberine, the 22AG sequence adopts an antiparallel conformation, which is also pressure stable, i.e., the complex is also densely packed. In this case, the Δ*V*_r_ value of −2.8 mL mol^−1^ includes the volume change for the conformational transition from the hybrid-1 to the antiparallel conformation, which can be estimated to be ~37 mL mol^−1^ [33]. Thus, the “true” binding volume would be around −39.8 mL mol^−1^. The overall negative volume change upon binding may be largely due to release of packing defects of the quadruplex structure upon binding of the ligand, which is reflected in a high pressure stability of the complex.

The binding scenario for RG-1 is entirely different. In both salt solutions, the reaction volume is more negative compared to that observed for 22AG (about −13 mL mol^−1^). In the presence of K^+^, the RG-1 sequence adopts a parallel conformation in both the uncomplexed and the complexed form, which is pressure stable up to about 2 kbar, pointing to a compact structure devoid of void volume in both cases. As no significant conformational changes were observed up binding of berberine, the reaction volume is expected to essentially reflect the volume change upon binding. The negative binding volume could result from an increase of hydration (electrostriction effect), which is less likely, or from the release of packing defects of the G4Q structure upon binding. The negative binding volume and the negative entropy change observed (*T*Δ*S*°_b_ = −66.9 kJ mol^−1^) would be consistent with a release of packing defects upon ligand binding, which leads to a more compact and less flexible structure, being also consistent with an increase of *E*_rel_ upon ligand binding. The thermodynamic parameters of complex formation in the presence of Na^+^ are quite different. As previously reported, the RG-1 structure in Na^+^ solution is not exclusively folded in a parallel conformation; other conformational states (unfolded or partially folded) are populated as well. For this system, the entropy change upon berberine binding is positive, indicating a significant release of structured water. Since a decrease of hydration should contribute positively to Δ*V*_r_, other explanations need to be invoked to explain the overall negative value of Δ*V*_r_. Most likely, again, compaction of the RG-1 structure upon ligand binding leads to the observed negative value of Δ*V*_r_, which would also be consistent with the small changes seen in the CD spectra upon ligand binding.

## 4. Materials and Methods

### 4.1. Materials

The sequences forming the quadruplexes were purchased from GenScript (Leiden, The Netherlands). In this study, two quadruplex sequences were used: the human telomeric DNA with the sequence GGGTTAGGGTTAGGGTTAGGG (22AG) and the SARS-CoV-2 RNA with the sequence GGCUGGCAAUGGCGG (RG-1). The labeled sequences employed for the FRET experiments were labeled with carboxyfluorescein (FAM) in 5′ and 5-(and-6)-carboxytetramethylrhodamine (TAMRA) in position 3′ and were purchased from GenScript. Tris(hydroxymethyl)aminomethane hydrochloride (Tris-HCl), dimethyl sulfoxide (DMSO), and the salts sodium chloride (NaCl) and potassium chloride (KCl) were purchased from Sigma Aldrich Chemicals (Merck, Darmstadt, Germany). The fluorescent compound berberine chloride (Berb) was also purchased from Sigma Aldrich Chemicals (Merck, Darmstadt, Germany). All the chemicals were used without further purification.

### 4.2. Sample Preparation

The pressure stable Tris-HCl buffer was used at the concentration of 30 mM, pH 7.4, with the addition of 100 mM NaCl or KCl. Concentrated stock solutions of 22AG and RG-1 quadruplexes were prepared by dissolving them in the appropriate Tris-HCl buffer (with NaCl or KCl). Their concentrations were determined by using the extinction coefficients *ε*(260) of 228,500 M^−1^ cm^−1^ for 22AG and 143,800 M^−1^ cm^−1^ for RG-1, respectively [44,45]. The absorbance was evaluated at the temperature of 90 °C (where the sequences are completely unfolded) by using a Shimadzu UV-1800 spectrometer (Shimadzu Corporation, Tokyo, Japan) and employing a 1-cm quartz cuvette (volume of 3 mL). The concentrated stock solution of berberine (50 mM) was prepared in dimethyl sulfoxide (DMSO). Its concentration was evaluated by diluting it in water and measuring its absorbance at 421 nm, using the extinction coefficient of 4209 M^−1^ cm^−1^ [46]. Since DMSO can affect nucleic acid structures [47], its concentration never exceeded 0.6 vol% in all experiments.

### 4.3. UV/Vis Spectrometry

UV/Vis spectra of berberine solutions, at a concentration of 250 µM, in the absence and in the presence of 22AG or RG-1 were recorded by means of a UV-1800 spectrometer from Shimadzu Corporation (Tokyo, Japan) at the temperature of 25 °C using a 0.3 cm path length quartz cuvette (final volume 60 µL) in the range 350–550 nm. The concentration of the G4Qs were varied between 0 and 150 µM. Tris-HCl buffer, 30 mM, pH 7.4, with the addition of 100 mM NaCl or KCl was used.

### 4.4. Steady-State Fluorescence Spectroscopy

The formation of the complex between berberine and the G4Qs (22AG and RG-1) was followed by means of fluorescence spectroscopy since berberine is almost non-fluorescent when dissolved in solution, whereas a strong fluorescence enhancement is observed upon binding to G4Qs. All the titration experiments were performed by using a K2 fluorimeter from ISS (Champaign, IL, USA). Briefly, a solution of berberine, at the concentration of 15 µM, was titrated with a solution of 22AG or RG-1 in the concentration range 0–40 µM. The excitation wavelength was set to 443 nm and the fluorescence intensity was collected at 536 nm, which is the position of the emission maximum in all cases. The monochromator slits were set to 16 nm for both the excitation and the emission. The temperature was set to 25 °C. For the high hydrostatic pressure (HHP) experiments, a high-pressure cell system from ISS and quartz cuvettes were used. The pressure was controlled by means of a manual pump and water was used as pressurizing fluid. The pressure points explored were 1, 500, 1000, 1500, and 1900 bar. Briefly, the samples were loaded in a pressure resistant quartz cuvette, sealed with DuraSeal™ laboratory stretch film and placed into the high-pressure cell. To determine the binding constant, *K*_b_, a Δ*F* was plotted vs. the total 22AG or RG-1 concentration. Here, Δ*F* = *F* − *F*_0_, where *F* and *F*_0_ are the fluorescence intensities of berberine in the presence and in the absence of G4Qs, respectively. The data were fitted with an *n* equivalent and independent binding sites model. For modeling details, please refer to [30].

### 4.5. Job’s Plot

In order to evaluate the stoichiometry of the complex formed, the continuous variation method (or Job’s plot) was employed [26,48]. To this end, a series of solutions of berberine and G4Qs (22AG or RG-1) were prepared in both media (Na^+^ and K^+^ containing buffers). The total concentration ([Berb] + [G4Q]) was 80 µM, 60 µM, 60 µM, and 50 µM for 22AG/Berb in the presence of NaCl, for 22AG/Berb in the presence of KCl, for RG-1/Berb in the presence of NaCl, and for RG-1/Berb in the presence of KCl, respectively. The mole fraction of berberine, *x*_Berb_, was varied between 0.1 and 1.0. Fluorescence intensities were recorded at 536 nm upon excitation at 443 nm. All the experiments were performed at *T* = 25 °C and ambient pressure (1 bar). For the system 22AG/Berb in NaCl buffer, the experiments were also performed at high pressure (500, 1000, 1500, and 1900 bar).

### 4.6. Fluorescence Resonance Energy Transfer

Fluorescence resonance energy transfer (FRET) experiments were performed in order to determine the conformational behavior of the G4Qs under pressure (pressure range: 1–2000 bar), in the absence and in the presence of berberine. For these experiments, labelled 22AG and RG-1 were used. Briefly, solutions of labelled 22AG at the concentration of 2 µM in the absence and in the presence of 200 µM berberine were prepared. The excitation wavelength was set to 490 nm. The fluorescence emission was recorded in the range 500–650 nm. The experiments were carried out in 30 mM Tris–HCl, pH 7.4, and in the same buffer containing 100 mM NaCl or 100 mM KCl at *T* = 25 °C. The relative FRET efficiency, *E*_rel_, was calculated by using the relation *E*_rel_ = *I*_A_/(*I*_A_ + *I*_D_) [49]. In this equation, *I*_D_ and *I*_A_ are the fluorescence intensities at the maximum of the donor (FAM) and the acceptor (TAMRA), respectively.

### 4.7. Circular Dichroism Spectroscopy

Circular dichroism (CD) spectroscopy was employed to study the conformations adopted by 22AG and RG-1 in the different solutions and upon binding to berberine at ambient pressure. CD spectra were recorded by means of a Jasco J-715 spectropolarimeter (Jasco Corporation, Tokyo, Japan) at the temperature of 25 °C. The path length of the cuvette was 0.1 cm. The spectra were acquired in the spectra range between 230 nm and 330 nm. The reported spectra represent the results of 3 accumulations. The concentrations of 22AG and RG-1 were 30 µM, and the concentration of berberine was 300 µM. The following instrumental parameters were used: scan rate 50 nm min^−1^, band width 5 nm band, and response time 2 s. For each sample, a blank (buffer with and without berberine) was recorded and subtracted. All the reported spectra were first converted from millidegrees to absorbance. Then, the absorbance was normalized per single strand concentration and path length, yielding Δ*ε* (M^−1^ cm^−1^).

### 4.8. Isothermal Titration Calorimetry

Isothermal titration calorimetry (ITC) was performed to measure the standard enthalpy of binding (Δ*H*°_b_). The measurements were carried out by means of a Nano ITC-III from TA Instruments (New Castle, DE, USA). All the experiments were carried out at *T* = 25 °C. Briefly, for the experiments carried out in the presence of KCl, a solution of 70 µM 22AG was injected into the calorimeter vessel (volume of 961 µL) where a solution of 80 µM berberine was placed. To determine the enthalpy of binding of 22AG in the presence of NaCl or RG1 in the presence of NaCl or KCl, a 150 µM berberine solution was titrated with an oligonucleotide solution at the concentration of 100 µM. In all cases, the injection volume was 15 µL. To ensure appropriate mixing of the solutions, a stirring speed of 250 rpm was applied. The heat of dilutions of the G4Qs were determined by injecting the G4Q solutions into the calorimeter vessel where the appropriate buffer without berberine was placed. The heat peaks obtained from the titration experiments were integrated by means of the NanoAnalyze software (TA Instruments, New Castle, DE, USA, software version: 3.11.0) supplied with the instruments. The heat peaks were normalized by the moles of bound G4Qs and are reported in kJ mol^−1^.

## 5. Conclusions

We demonstrated that berberine, a broad-spectrum antiviral compound, which is also effective against SARS-CoV-2, binds effectively to both DNA and viral RNA quadruplexes via π-π stacking and possibly also via cation-π and cation-lone pair (-CO) interaction of the positively charged N7 atom of berberine with the G-quadruplex plane. The reported data show that berberine interacts readily with both quadruplexes and that two berberine molecules are bound to one G4Q in both cases. Considerable differences were observed for the interaction of berberine with DNA-G4Q 22AG and RNA-G4Q RG-1. The two G-quadruplex structures adopt different topologies, and we found that the strength of interaction with the human telomeric DNA-G4Q 22AG sequence depends on the initial conformation of the nucleic acid, which is not the case for viral RNA RG-1. Next to changes in quadruplex structure for 22AG in KCl solution, minor local changes in the conformational dynamics were seen upon berberine binding. Generally, upon complex formation, a compaction of the G4Q structure is noticed and, where present, the population of (partially) unfolded states vanishes. The present study may be useful for the development of new antiviral drugs based on the berberine structure. Further, the results obtained may serve as a starting point for the development of modified berberine molecules that can bind to RG-1 with higher selectivity. To this end, further studies (e.g., a structural characterization by NMR and molecular dynamics simulations with atomic resolution) are needed to unravel the complex binding mechanism in more detail. These studies will be essential to highlight the differences in the binding mode of berberine with 22AG and to understand the molecular determinants that can be used to make berberine more selective for RG-1.

## Figures and Tables

**Table 1:** Thermodynamics parameters for the complex formation between berberine and 22AG and RG-1 at the indicated solution conditions, at the temperature of 25 °C and pressure of 1 bar.

System	*K*_b_/M^−1^ ∙ 10^5^	^1^ Δ*G*°_b_ /kJ mol^−1^	^2^ Δ*H*°_b_ /kJ mol^−1^	^3^ TΔ*S*°_b_ /kJ mol^−1^	^4^ Berb:G4Q
Berb/22AG in NaCl buffer	0.37 ± 0.09	−26.0 ± 0.6	−15.5 ± 2.8	10.5 ± 3.4	2:1
Berb/22AG in KCl buffer	5.9 ± 1.0	−32.9 ± 0.4	19.5 ± 3.8	52.4 ± 4.2	2:1
Berb/RG-1 in NaCl buffer	0.28 ± 0.03	−25.4 ± 0.3	75.9 ± 7.6	101.3 ± 7.2	2:1
Berb/RG-1 in KCl buffer	0.14 ± 0.03	−23.7 ± 0.4	−92.2 ± 12.0	−66.9 ± 8.8	2:1

^1^ The standard Gibbs (free) energy changes of binding were calculated using Δ*G*°_b_ = −*RT*ln(*K*_b_). ^2^ Obtained from ITC experiments. ^3^ The entropy changes upon binding were calculated from the relation *T*Δ*S*°_b_ = Δ*H*°_b_ − Δ*G*°_b_. ^4^ Stoichiometry of the complexes.

**Table 2:** Binding constants, *K*_b_, and stoichiometries for the complex formation between berberine and G4Qs (22AG and RG-1) for the indicated solution conditions and at pressures of 1, 500, 1000, 1500, and 1900 bar (*T* = 25 °C).

**Berb/22AG NaCl (100 mM)**			**Berb/22AG KCl (100 mM)**		
***p/*bar**	***K*_b_/M^−1^ ∙ 10^5^**	**^1^ Berb:G4Q**	***p/*bar**	***K*_b_/M^−1^ ∙ 10^5^**	**^1^ Berb:G4Q**
1	0.37 ± 0.09	2:1	1	5.9 ± 1.0	2:1
500	0.39 ± 0.07	2:1	500	6.6 ± 0.9	2:1
1000	0.44 ± 0.06	2:1	1000	6.9 ± 0.7	2:1
1500	0.43 ± 0.09	3:1	1500	7.1 ± 0.4	2:1
1900	0.50 ± 0.09	3:1	1900	7.3 ± 0.5	2:1
**Berb/RG-1 NaCl (100 mM)**			**Berb/RG-1 KCl (100 mM)**		
***p/*bar**	***K*_b_/M^−1^ ∙ 10^5^**	**^1^ Berb:G4Q**	***p/*bar**	***K*_b_/M^−1^ ∙ 10^5^**	**^1^ Berb:G4Q**
1	0.28 ± 0.03	2:1	1	0.14 ± 0.03	2:1
500	0.38 ± 0.05	2:1	500	0.17 ± 0.03	2:1
1000	0.49 ± 0.06	2:1	1000	0.23 ± 0.02	2:1
1500	0.63 ± 0.07	2:1	1500	0.31 ± 0.01	2:1
1900	0.77 ± 0.09	2:1	1900	0.40 ± 0.06	2:1

^1^ Stoichiometry of the complexes.

## Data Availability

The data presented in this study are available on request from the corresponding authors.

## Supplementary Materials

Supplementary materials can be found at https://www.mdpi.com/article/10.3390/ijms23105690/s1. Reference [50] is cited in the Supplementary Materials.
