# Supplementary material for: Binding Properties of RNA Quadruplex of SARS-CoV-2 to Berberine Compared to Telomeric DNA Quadruplex
Source: Int J Mol Sci. 2022 May 19;23(10):5690. doi: 10.3390/ijms23105690 (PMC9145931; doi:10.3390/ijms23105690)
Supplement: Supplementary file 1 [file ijms-23-05690-s001.zip › ijms-1701777-supplementary.pdf]

International Journal of
*Molecular Sciences*

MDPI

# Supplementary Information

## The method of continuous variation (Job's plot)

The method of continuous variation, also known as Job's plot, is a convenient method for the determination of the stoichiometry of complex formation. In this method, a series of solutions of the interacting partners is prepared such that their total concentration is kept constant, while varying their mole fraction. Then, of these solutions, any physical observable (e.g., the fluorescence intensity) that is directly correlated to the complex formation is followed. A plot of the physical observable *vs.* the mole fraction of one of the interacting partners is prepared. Abrupt changes in this plot indicates the stoichiometry of the complex. This method seems to be quite easy to apply and, thus, very attractive. However, the accuracy and the identification of the exact stoichiometry as well as the identification of multiple complexes present in solution have to be taken with care, as previously described in detail [1,2]. In particular, the shape of the plot and the position of the inflection point depend on the magnitude of the binding constant, $K_b$, and on the total concentration used, which is, in turn, limited by the technique applied. Here, we show that for the system 22AG-berberine in $Na^+$ containing buffer (which is characterized by a $K_b$ value of $3.65 \cdot 10^4$ $M^{-1}$ at $T = 25$ °C and $p = 1$ bar), the simulated Job's plot is in very good agreement with the experimental one and that the stoichiometry is 2:1 (Berb:22AG). In Fig. S1, the simulated Job's plot obtained by imposing a stoichiometry of 2:1 (Berb:22AG) and using the experimental value of $K_b$ with a total concentration of 80 µM is reported. In addition, the experimental Job's plot (black squares) is also shown. From the experimental Job's plot, an inflection point at $x_{Berberine}$ of 0.6 is obtained. For a 1:1 and 2:1 (Berb:22AG) complex, the inflection points should be located at 0.5 and 0.67, respectively. However, since the Job's plot is dependent from the value of the binding constant and the total concentration used, for a 2:1 complex the inflection point is located at 0.6, as shown by the simulation. In fact, the experimental plot is in very good agreement with the theoretical one, supporting the conclusion that the complex stoichiometry is 2:1.

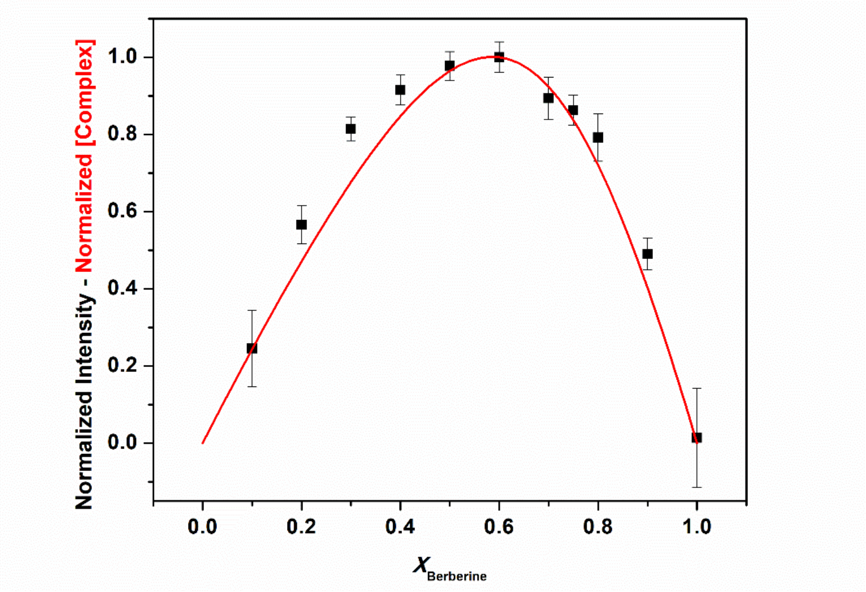


**Figure S1.** Superimposition of the simulated Job´s plot (red line) and the experimental one (black squares) for the 22AG-berberine system in NaCl containing buffer. The simulated plot was obtained using the experimental value of $K_b = 3.65 \cdot 10^4$ $M^{-1}$ and the total concentration of 80 µM used in the experiment and reported in Fig. 2 of the main text.

To further support to above conclusion, Job's plot simulations obtained using the experimental parameters ($K_b$ and the total concentration) but stoichiometries of 1:1, 2:1, 3:1, and 4:1 (Berb:22AG) are depicted in Fig. S2.

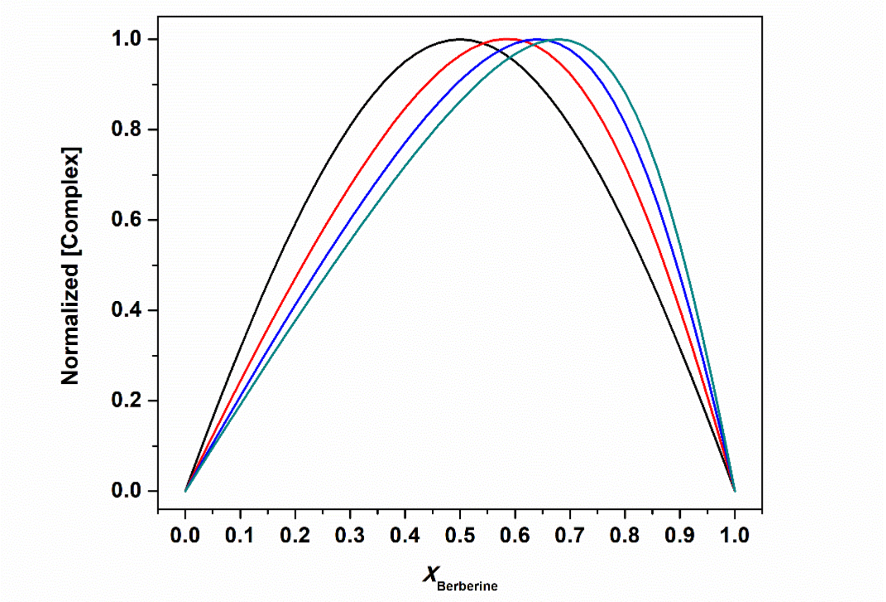


**Figure S2.** Simulated Job's plot for the 22AG-berberine system in the $Na^+$ containing buffer. The plots were obtained by using the experimental value of $K_b = 3.65 \cdot 10^4$ $M^{-1}$, a total concentration of 80 μM and imposing a stoichiometry (Berb:22AG) of 1:1 (black line), 2:1 (red line), 3:1 (blue line), and 4:1 (green line).

As reported in Fig. S2, for a 1:1 (Berb:22AG) complex, the inflection point is centred at about $x_{Berberine}$ = 0.5. Instead, when a 2:1 complex is formed, as reported above, $x_{Berberine}$ is around 0.6 (in an ideal case, it should be at 0.67). For a 3:1 complex, the inflection point is at 0.65, whereas it should be at 0.75 in an ideal case. Finally, for a 4:1 (Berb:22AG) complex, $x_{Berberine}$ is located at 0.69 (in an ideal case, it should be at 0.80). These observations validate our conclusion that the stoichiometry of the complex is 2:1 and cannot be higher.

Finally, we are showing the dependence of the Job's plot from the value of $K_b$ and the total berberine concentration. The simulated Job´s plots are shown in Fig. S3.

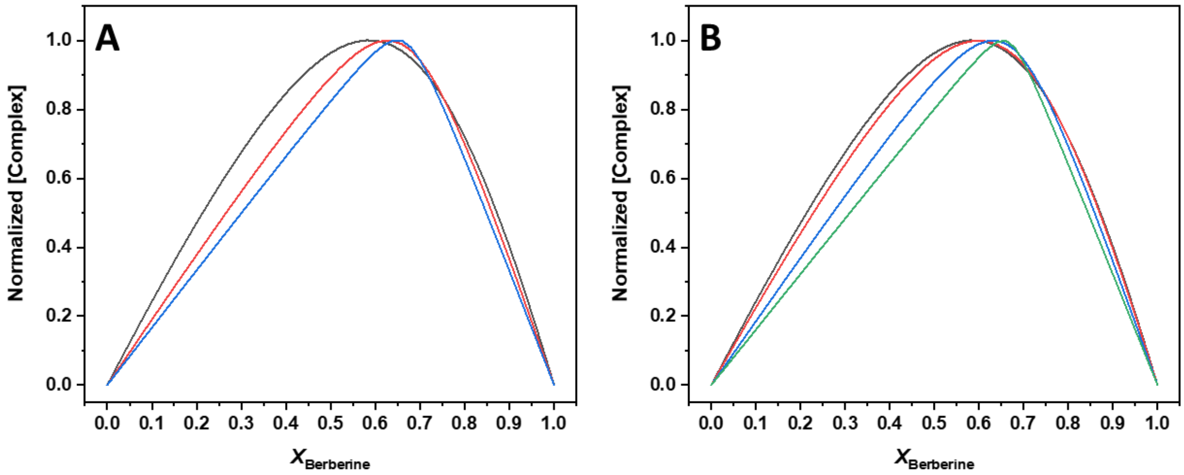

**Figure S3.** Job´s plot simulation for the 22AG-berberine system in the $Na^+$ containing buffer. Panel A depicts how the Job´s plot depends on the total berberine concentration. The black line represents the plot obtained using the obtained $K_b$ of $3.65 \cdot 10^4$ $M^{-1}$ and the total berberine concentration ([Berb]+[22AG]) of 80 µM used in the experiment. The red and blue curves reported the plots obtained using the same $K_b$, but a total berberine concentration of 500 µM and 3000 µM, respectively. Panel B shows the dependence of the Job´s plot on the value of $K_b$. The black line represents the plot obtained using the obtained $K_b$ of $3.65 \cdot 10^4$ $M^{-1}$ and the total concentration of 80 µM used in the experiment. In red, blue, and green are shown the plots obtained using the same total berberine concentration of 80 µM, but a $K_b$ value of $6.0 \cdot 10^4$ $M^{-1}$, $3.0 \cdot 10^5$ $M^{-1}$, and $3.0 \cdot 10^6$ $M^{-1}$, respectively. On the *y*-axis, the normalized total complex concentration is reported. The mole fraction of berberine is shown on the *x*-axis. In all cases, a stoichiometry of 2:1 Berb:22AG was imposed.

When the Job's plot for the 22AG-berberine system in NaCl buffer is simulated using the experimental values of $K_b = 3.65 \cdot 10^4$ $M^{-1}$ and the total berberine concentration, $c_{Berberine} + c_{22AG}$, of 80 µM, the black plots reported in Fig. S3 were obtained, imposing a stoichiometry of 2 (2 berberine molecules bound to one 22AG). As it is evident from simulations, the inflection point, which ideally should be located at $x_{Berberine} = 0.67$, is positioned around 0.6, as observed in the experiment (see Fig. S1). This is due to the low value of the binding constant and the particular total concentration used, which is determined by the experimental technique chosen. For instance, in fluorescence spectroscopy the concentration of the fluorophore berberine should not exceed 0.1 absorbance units at the excitation wavelength. Indeed, if the total concentration is increased (Fig. S3, panel A), a shift of the inflection point passing the true stoichiometry is observed. In the simulated cases, at a total concentration of 500 µM, the inflection point is located at $x_{Berberine} = 0.62$. At a total concentration of 3000 µM, the inflection point is around 0.66, close to the expected value for a 2:1 stoichiometry. Of note, with increasing concentration, the Job's plot becomes sharper, reducing the uncertainty on the position of the inflection point. The dependence of the shape and inflection point position on the value of the $K_b$ is shown in panel B of Fig. S3. It is clear that, holding the total concentration $c_{Berberine} = 80$ µM used for the experiment, an increasing $K_b$ value leads to a sharper plot and a shift of the inflection point towards a higher value of $x_{Berberine}$. For example, a $K_b$ as high as $3.0 \cdot 10^6$ $M^{-1}$ leads to an inflection point at 0.66, which is close to the true 2:1 stoichiometry. In conclusion, the position of the inflection point and the shape of the Job's plot depend on the magnitude of the binding constant and the total concentration of the interacting partners chosen. Hence, theoretical modelling of Job's plot data are needed for determining the stoichiometry of the complex precisely.

**FRET and CD spectra of the RG-1 quadruplex in neat buffer solution**

To assess the impact of the dual labelling procedure on the structure of RG-1, FRET experiments in combination with CD spectroscopy were performed as a function of temperature in 30 mM Tris buffer, pH 7.4 (Figure S4).

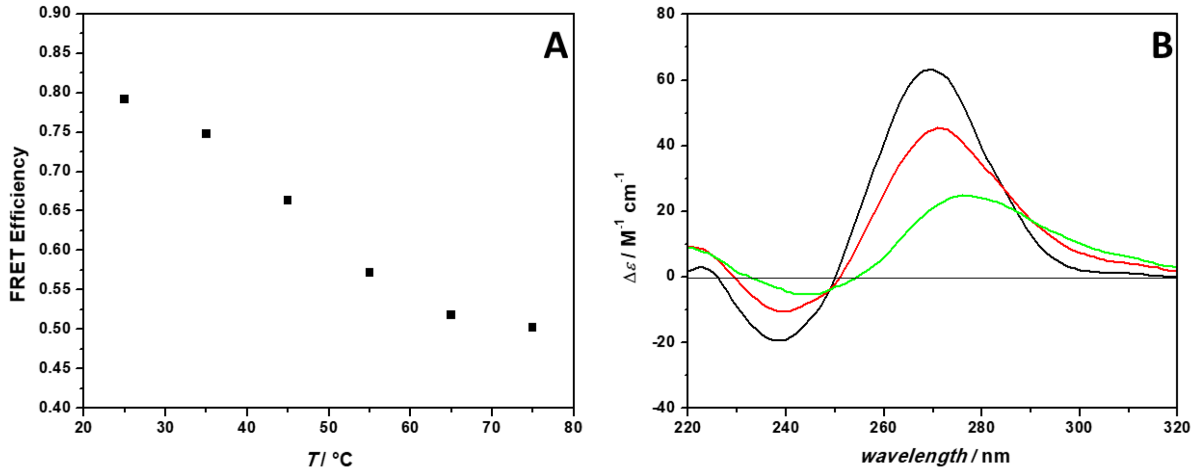


**Figure S4.** (**A**) Relative FRET efficiency *vs.* temperature for the dually labeled RG-1 sequence. Where not visible, the error bars are smaller than the symbol size. (**B**) Circular dichroism spectra of RG-1 at the selected temperatures of: 25 °C (black), 45 °C (red), and 75 °C (green). All the experiments were performed in 30 mM Tris-HCl buffer, pH 7.4.

In neat buffer (no salts), RG-1 is already able to fold in a parallel quadruplex structure at $T = 25$ °C, as previously reported [3]. Indeed, the FRET efficiency calculated at 25 °C is similar to that calculated in the presence of $Na^+$ and $K^+$. In addition, the CD spectrum (black spectrum in Fig. 2, panel B) shows the features characteristic of a parallel conformation, i.e., a positive band around 270 nm and a weak negative band around 240 nm. Upon increasing the temperature, the FRET efficiency gradually decreased, reaching a value around 0.50 at 75 °C. At the same time, a clear change in the CD spectrum was observed with temperature. At 45 °C, the intensities of both the positive and negative bands decreased, coupled with a small shift towards higher wavelengths. This change is even more pronounced at 75 °C where the RG-1 seems to be completely unfolded. From these data we can infer that the two fluorophores have no significant impact on the conformational dynamics of RG-1 and, thus, they can be used to probe conformational changes of this sequence.

## Job's plot for the Berb/22AG complex in the presence NaCl at high pressures

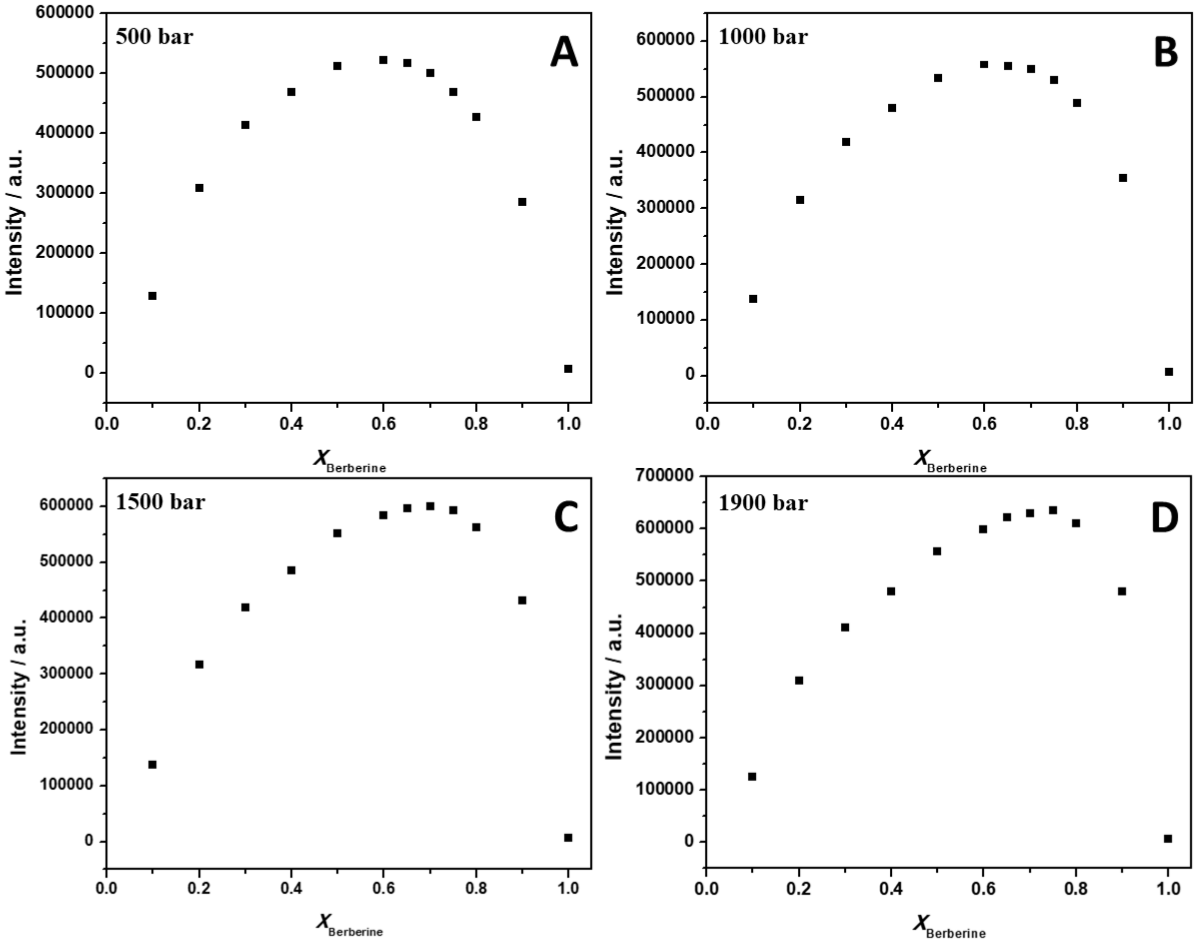


**Figure S5.** Job's plot for the berb/22AG complex formation in 30 mM Tris-HCl buffer, pH 7.4, and 100 mM NaCl at the pressures of 500 bar (**A**), 1000 bar (**B**), 1500 bar (**C**), and 1900 bar (**D**). The total concentration ([Berb]+[GQs]) was 60 µM. The excitation wavelength was set at 443 nm. The fluorescence intensity was recorded at the maximum of emission (536 nm). Where not visible, the error bars are smaller than the symbol size.

## References

1. Brynn Hibbert, D.; Thordarson, P. The Death of the Job Plot, Transparency, Open Science and Online Tools, Uncertainty Estimation Methods and Other Developments in Supramolecular Chemistry Data Analysis. *Chem. Commun.* **2016**, *52*, 12792–12805. https://doi.org/10.1039/C6CC03888C.
2. Ulatowski, F.; Dąbrowa, K.; Bałakier, T.; Jurczak, J. Recognizing the Limited Applicability of Job Plots in Studying Host–Guest Interactions in Supramolecular Chemistry. *J. Org. Chem.* **2016**, *81*, 1746–1756. https://doi.org/10.1021/acs.joc.5b02909.
3. Bezzi, G.; Piga, E.J.; Binolfi, A.; Armas, P. CNBP Binds and Unfolds In Vitro G-Quadruplexes Formed in the SARS-CoV-2 Positive and Negative Genome Strands. *IJMS* **2021**, *22*, 2614. https://doi.org/10.3390/ijms22052614.
